# Longitudinal Follow-Up of the Immunity to SARS-CoV-2 in Health Care Workers in Argentina: Persistence of Humoral Response and Neutralizing Capacity after Sputnik V Vaccination

**DOI:** 10.1128/msphere.00662-22

**Published:** 2023-04-18

**Authors:** Eliana F. Castro, Julián Acosta, Lucía Moriena, Mayra Rios Medrano, Malena Tejerina Cibello, Eduardo Codino, Miguel Ángel Taborda, Diego E. Álvarez, Ana Laura Cavatorta

**Affiliations:** a Instituto de Investigaciones Biotecnológicas (IIBIO), Universidad Nacional de San Martín (UNSAM) – Consejo Nacional de Investigaciones Científicas y Técnicas (CONICET), Buenos Aires, Argentina; b Instituto de Virología e Innovaciones Tecnológicas (IVIT), Centro de Investigaciones en Ciencias Veterinarias y Agronómicas (CICVyA), Instituto Nacional de Tecnología Agropecuaria (INTA, CONICET), Buenos Aires, Argentina; c Instituto de Biología Molecular y Celular de Rosario-CONICET, Santa Fe, Argentina; d Facultad de Ciencias Bioquímicas y Farmacéuticas, Universidad Nacional de Rosario, Santa Fe, Argentina; e Centro de Tecnología en Salud Pública (CTSP), Facultad de Ciencias Bioquímicas y Farmacéuticas, Universidad Nacional de Rosario, Santa Fe, Argentina; f Escuela de Bio y Nanotecnologías (EByN), Universidad Nacional de San Martín, Buenos Aires, Argentina; The University of Texas Southwestern Medical Center

**Keywords:** SARS-CoV-2, Sputnik V, humoral immune response, hybrid immunity, vaccination

## Abstract

SARS-CoV-2 vaccine protection has encountered waning of immune response and breakthrough infections. The hybrid immune response generated by the combination of vaccination and infection was shown to offer higher and broader protection. Here, we present a seroprevalence study of anti-SARS-CoV-2 spike/RBD IgG in 1,121 health care workers immunized with Sputnik V and a follow-up of humoral response at 2 and 24 weeks postvaccination (wpv), including neutralizing antibody response (NAT) against ancestral, Gamma, and Delta variants. The first seroprevalence study showed that among 122 individuals with one dose, 90.2% were seropositive versus 99.7% seropositivity among volunteers with the complete two-dose regimen. At 24 wpv, 98.7% of the volunteers remained seropositive, although antibody levels decreased. IgG levels and NAT were higher in individuals that had acquired COVID-19 previous to vaccination than in naive individuals at 2 and 24 wpv. Antibody levels dropped over time in both groups. In contrast, IgG levels and NAT increased after vaccine breakthrough infection. At 2 wpv, 35/40 naive individuals had detectable NAT against SARS-CoV-2 Gamma and 6/40 against Delta. In turn, 8/9 previously infected individuals developed a neutralizing response against SARS-CoV-2 Gamma and 4/9 against Delta variants. NAT against variants followed a trajectory similar to NAT against ancestral SARS-CoV-2, and breakthrough infection led to an increase in NAT and complete seroconversion against variants. In conclusion, Sputnik V-induced humoral response persisted at 6 months postvaccination, and hybrid immunity induced higher levels of anti-S/RBD antibodies and NAT in previously exposed individuals, boosted the response after vaccination, and conferred wider breadth of protection.

**IMPORTANCE** Since December 2020, Argentina has begun a mass vaccination program. The first vaccine available in our country was Sputnik V, which has been approved for use in 71 countries with a total population of 4 billion people. Despite all the available information, there are fewer published studies on the response induced by Sputnik V vaccination compared to that of other vaccines. Although the global political context has paralyzed the verification by the WHO of the efficacy of this vaccine, our work aims to add new clear and necessary evidence to Sputnik V performance. Our results contribute to general knowledge of the humoral immune response developed by vaccines based on viral vector technology, highlighting the higher immune protection conferred by hybrid immunity and reinforcing the importance of completing vaccination schedules and booster doses to maintain adequate antibody levels.

## INTRODUCTION

Countries achieving high coverage of COVID-19 vaccination have benefited from minimizing deaths, severe disease, and overall disease burden. Mass roll-out of vaccination in Argentina started on 29 December 2020. The Sputnik V vaccine was the first vaccine to become available and was approved under the emergency use authorization procedure. First, target population was defined according to risk to exposure, prioritizing health care workers.

The Sputnik V vaccine comprises two vector components, recombinant adenovirus type 26 (rAd26) and recombinant adenovirus type 5 (rAd5), both of which carry the gene for SARS-CoV-2 full-length glycoprotein S of the prototypic Wuhan lineage. Both components were developed and manufactured by N. F. Gamaleya National Research Centre for Epidemiology and Microbiology (Moscow, Russia) (1, 2). Until February 2023, Sputnik V was approved for use in 71 countries, mainly in South America, Africa, and Asia, comprising a total population of 4 billion people (3). However, it has not been yet approved by the World Health Organization.

Waning of the antibody response against SARS-CoV-2 over time after vaccination has been well documented for the different vaccine platforms and, together with emergence of variants of concern (VOCs) associated with higher incidence of breakthrough infections, has hampered the control of the pandemic (4, 5). For Sputnik V vaccine, multiple reports have shown that anti-S/RBD antibody levels decreased over a period of approximately 6 months after complete vaccination (two doses with a 21-days interval between doses) (6 to 10). However, data on maintenance of seropositivity is divergent. Although seroprevalence after 2 to 3 weeks after second dose is generally 97 to 99%, some works showed that seropositivity decreased over a 6-month period to 83% (6) and even to 31% (11), whereas some others reported a maintenance of seropositivity in around 95% of the analyzed cohort (7, 9, 12).

Neutralizing response exerted by individuals vaccinated with Sputnik V was previously assessed mainly against ancestral SARS-CoV-2 in pseudovirus-based assays (9 to 11) and ELISA-based assays (7, 8). Previous longitudinal analysis of neutralizing antibodies performed up to at least 6 months after vaccination, showed a decrease of neutralizing titers over time (9, 10). Moreover, with the evolution of the pandemic, new SARS-CoV-2 variants emerged, and virus neutralization and epidemiological efficacy of vaccines drop against variants of concern (VOCs). As for other vaccine platforms, neutralizing antibodies exerted by Sputnik V vaccination also showed a reduced neutralizing potency against VOCs (9 to 11, 13, among others).

Hybrid immunity resulting from SARS-CoV-2 infection either prior to or after COVID-19 vaccination has been associated with broader protection, higher levels of neutralizing antibodies, and greater protection against symptomatic infection compared to immunity after vaccination or infection alone (14, 15). It was previously shown that total (6, 10, 11, 16, 17) and neutralizing (10, 11, 16) antibody response after Sputnik V vaccination is enhanced in previously infected individuals.

Regarding Sputnik V vaccine, there is less information available than for vaccines already approved by WHO. Our research aims to add new evidence and descriptive data concerning the quality and durability of the IgG and neutralizing immune response after Sputnik V vaccination. We performed a comprehensive follow-up study of total IgG and neutralizing antibodies against ancestral SARS-CoV-2, and Gamma and Delta VOCs in a cohort of health care workers fully vaccinated with Sputnik V. To gain insight into hybrid immunity, we analyze this cohort discriminating individuals as never infected, previously infected, and with vaccine breakthrough infections.

## RESULTS

### Study design.

**FIG 1:**
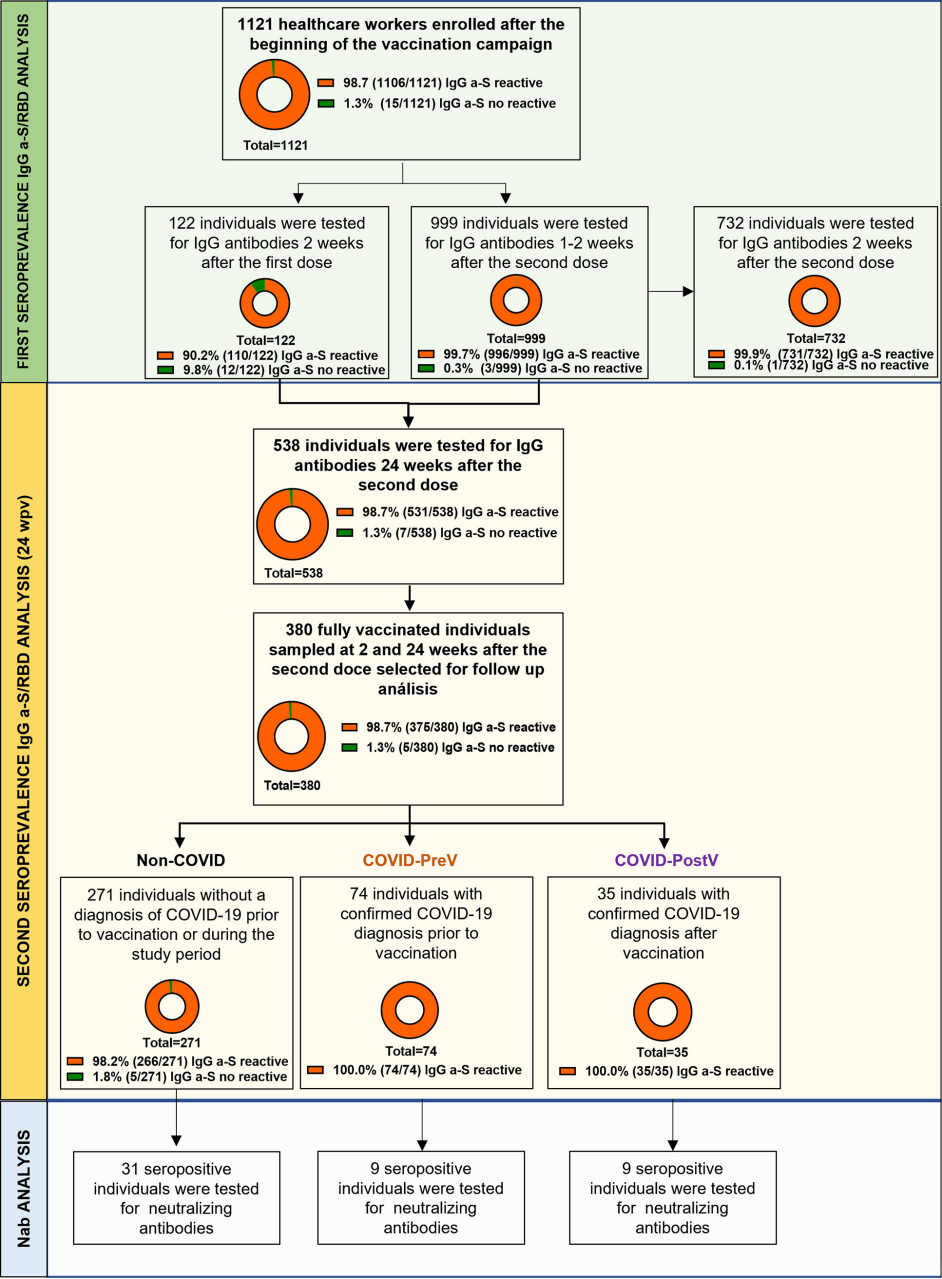
Design and main seropositivity results of anti-S/RBD SARS-CoV-2 IgG seroprevalence study. Seroprevalence study was carried out in a cohort of 1,121 individuals vaccinated with Sputnik V. First, the humoral immune response of individuals having received one (2 weeks after first dose, *n* = 122) or two doses (1 to 2 weeks after second dose, *n* = 999) after the beginning of the vaccination campaign was evaluated. A second seroprevalence study was carried out at 24 weeks after completion of the two-doses vaccination schedule (24 wpv) and included 538 fully vaccinated individuals from the initial cohort. Three hundred and eighty individuals with paired samples obtained both at 2 and 24 wpv were selected for further analysis and divided according to their COVID status (i) without a diagnosis of COVID-19 prior or during the study period (*n* = 271; Non-COVID); (ii) with confirmed COVID-19 diagnosis prior to the start of the vaccination schedule (COVID-PreV; *n* = 74); and (iii) with breakthrough infections (confirmed COVID-19 diagnosis between 2 and 24 wpv, COVID-PostV; *n* = 35). Finally, 49 of these individuals were selected for neutralizing antibodies (Nab) analysis.

A total of 1,121 health care workers vaccinated with the Sputnik V vaccine in Argentina between December 2020 and March 2021 at Hospital Provincial del Centenario, Rosario, Santa Fe, were enrolled in this study. Participants’ ages ranged from 21 to 78 years (median 42 years), and 725 (64.7%) were females. Vaccination with Sputnik V consisted of two doses with an interval between doses of 21 days. We carried out a first seroprevalence study between January and March 2021 in 1,121 individuals who were immunized with at least one dose of Sputnik V: 122 individuals were tested 2 weeks after the first dose, and 999 individuals were tested after the second dose, 732 of which were tested 2 weeks after completing the vaccination schedule (2 wpv). After 6 months (24 wpv), a new seroprevalence analysis was performed in 538 fully vaccinated volunteers. Among them, 380 individuals, who had been also initially tested at 2wpv, were selected for further analysis. To analyze humoral response in the context of hybrid immunity, this cohort was differentiated into individuals: (i) without a diagnosis of COVID-19 prior or during the study period (*n* = 271) (non-COVID, naive); (ii) with confirmed COVID-19 diagnosis prior to the start of the vaccination schedule (COVID-PreV) (*n* = 74); and (iii) with breakthrough infections (confirmed COVID-19 diagnosis between 2 and 24 wpv, COVID-PostV) (*n* = 35). COVID-19 diagnosis was confirmed by antigen tests or PCR. From this cohort of 380 individuals, 49 anti-S/RBD IgG seropositive individuals were selected to assess the neutralizing antibody response both at 2 and 24 wpv (Fig. 1).

### Seroprevalence studies.

Results of a first seroprevalence study carried out after the beginning of the vaccination campaign showed that 98.7% of the total cohort developed specific anti-S/RBD IgG antibodies (Fig. 1). At the time, 122 individuals had received one dose of Sputnik V, and 90.2% (110/122) were seropositive. Nine hundred and ninety-nine volunteers that had received the complete vaccination regimen were tested at 1 to 2 weeks after the second dose and showed 99.7% (996/999) seropositivity (Fig. 1). In particular, the 732 individuals that were analyzed at 2 weeks after completion of the vaccination schedule showed 99.9% (731/732) of seropositivity. We further assessed the response to complete vaccination by monitoring specific IgG antibodies at 24 wpv. Five hundred and thirty-eight individuals attended the second seroprevalence study, and 98.7% (531/538) were seropositive. A follow-up analysis of the humoral response after Sputnik V vaccination was performed in 380 fully vaccinated individuals that attended the study both at 2 and 24 wpv. All individuals of this subcohort were seropositive at 2 wpv, and 98.7% still presented specific IgG anti-S/RBD antibodies at 24 wpv (Fig. 1).

**FIG 2:**
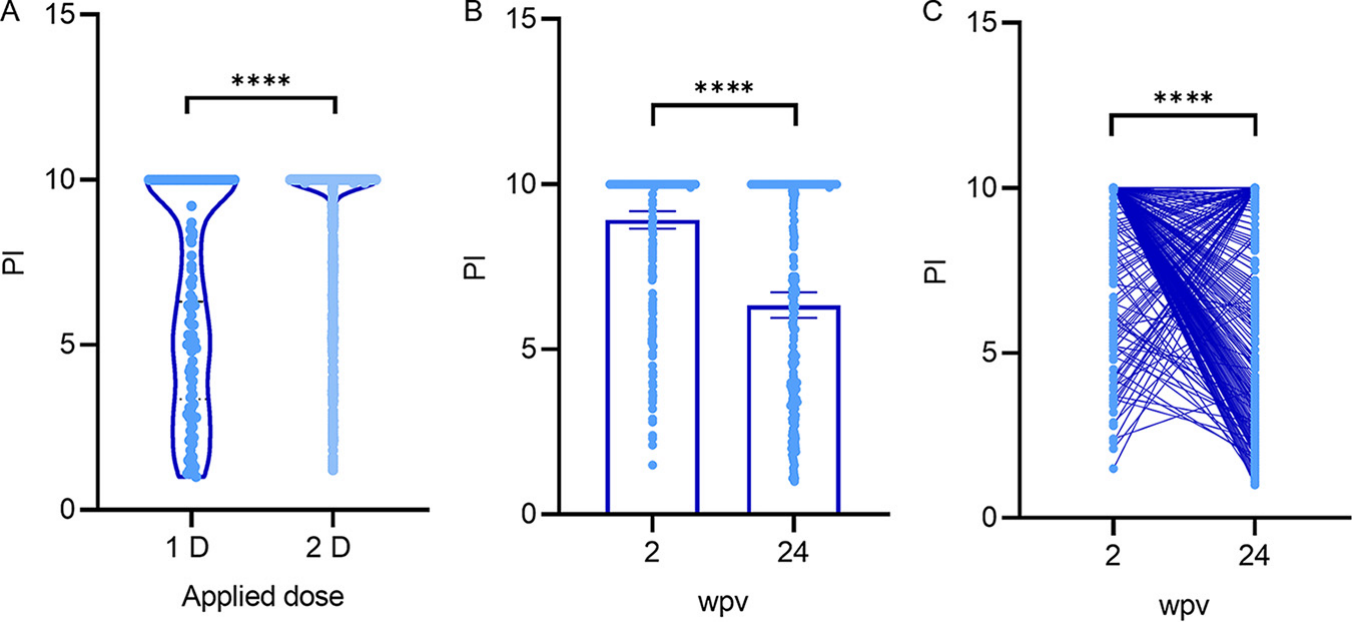
Total IgG antibody levels against Spike/RBD of SARS-CoV-2 after Sputnik V vaccination. The levels of IgG antibodies against the ectodomain of the original Wuhan spike protein and RBD were measured by COVIDAR IgG ELISA, and quantification was carried out calculating the PI values (range from 1 to 10) according to manufacturer’s instructions. (A) Serum samples were collected 2 weeks after application of the first dose (1D) (*n* = 122) and 1 to 2 weeks after second dose (2D) (*n* = 999) of Sputnik V. (B) Antibody levels were evaluated in 380 individuals after 2 and 24 weeks after completion of the vaccination schedule (wpv). (C) Individual evolution of total IgG anti-Spike/RBD levels at 2 and 24 wpv. Individual PI values and geometric mean with 95%CI are shown. Mann-Whitney U test was used for analysis of the total IgG antibody levels. Wilcoxon matched-pair test was used for individual longitudinal analysis of the total IgG antibody levels. Statistical significance is shown with the following notations: ****, *P* < 0.0001.

The analysis of anti-S/RBD antibody levels showed significantly lower levels in individuals who received one dose than in those who received two doses of Sputnik V (Fig. 2A). On the other hand, follow-up data of 380 individuals showed that over a period of 6 months, anti-S/RBD IgG antibody levels declined significantly (Fig. 2B and C).

### Humoral response in the context of hybrid immunity.

**FIG 3:**
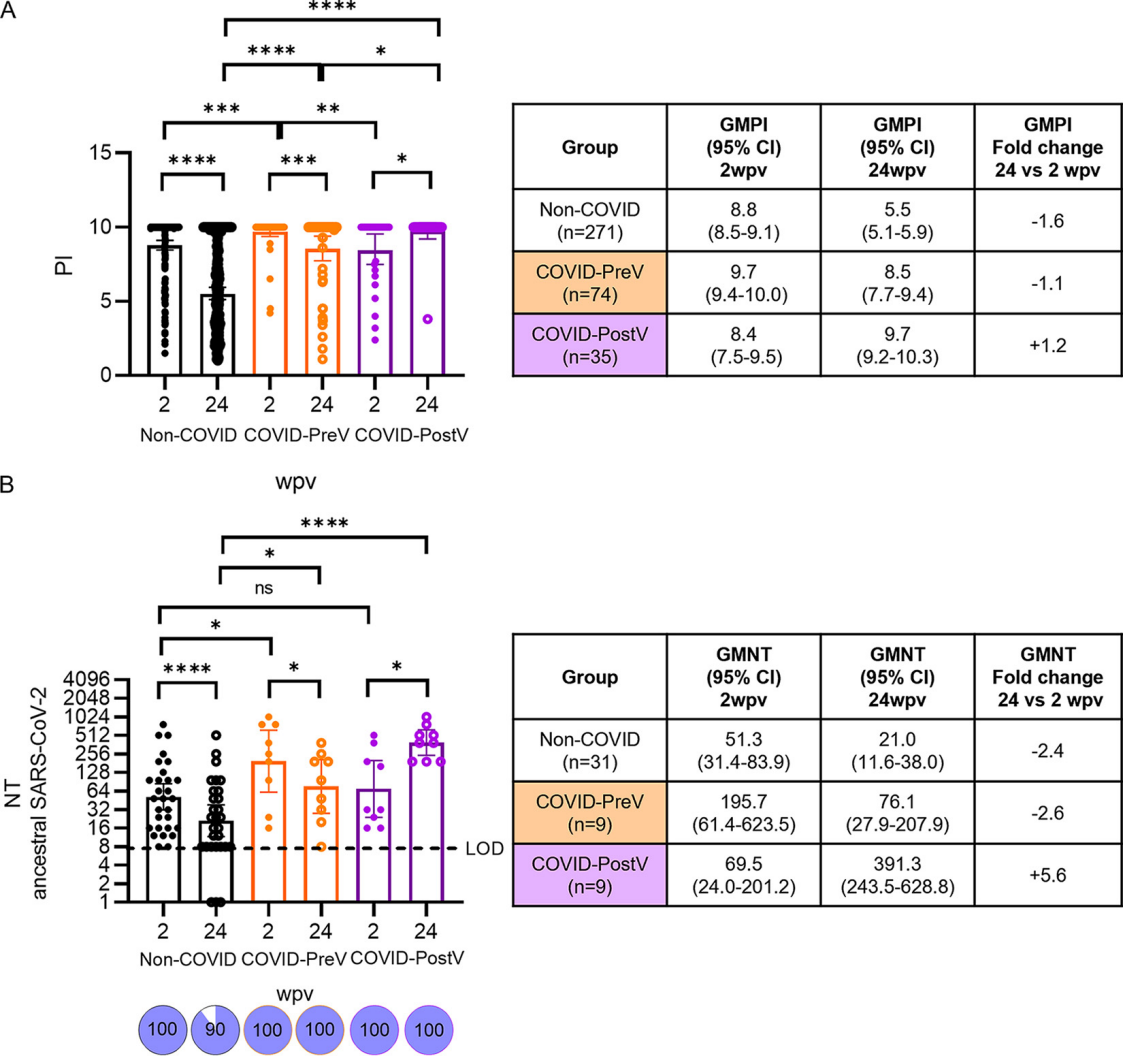
Evolution of total anti-Spike/RBD IgG and neutralizing antibody levels against ancestral SARS-CoV-2 after Sputnik V vaccination according to COVID-19 diagnosis. Sera collected at 2 and 24 wpv were obtained from a cohort of 380 individuals at 2 and 24 weeks after completion of the vaccination schedule (wpv) with Sputnik V. Cohort was differentiated into individuals (i) without a diagnosis of COVID-19 prior to vaccination or during the study period (Non-COVID; black dots); (ii) with confirmed COVID-19 diagnosis prior to the start of the vaccination schedule (COVID-PreV; orange dots); and (iii) with confirmed COVID-19 diagnosis after 2wpv (COVID-PostV; purple dots). (A) Anti-Spike/RBD IgG levels were evaluated in Non-COVID (*n* = 271), COVID-PreV (*n* = 74), and COVID-PostV (*n* = 35). Individual PI values and geometric mean with 95%CI are shown. (B) Neutralizing titers (NT) were defined as the highest serum dilution without any viral cytopathic effect on the monolayer using a replicating SARS-CoV-2. NT were evaluated in Non-COVID (*n* = 29); COVID-PreV (*n* = 9); and COVID-PostV (*n* = 9). Individual NT values and geometric mean with 95%CI are shown. Samples with NT below 8 (limit of detection, LOD) were set at 1 to calculate GMNTs. Pie charts illustrate the percentage of samples that resulted positive for neutralizing antibodies (NT ≥ 8). Tables next to each graph indicate the corresponding GM, 95%CI values, and GM fold change of 24 versus 2 wpv. Wilcoxon matched-pair test was used for longitudinal analysis of total IgG antibody levels and neutralizing titers. For nonpaired samples, the Mann-Whitney U test was used. Statistical significance is shown with the following notations: ****, *P* < 0.0001; ***, *P* < 0.001; **, *P* < 0.01; *, *P* < 0.05; ns, not significant.

Hybrid immunity is formed in individuals receiving a COVID-19 vaccine and experiencing SARS-CoV-2 infection before or after vaccination, and it is reported to induce higher antibody levels (14, 15). To evaluate humoral immune response in the context of hybrid immunity following Sputnik V vaccination, the cohort of 380 individuals was differentiated into individuals that did not acquire an infection (Non-COVID, *n* = 271); individuals that had acquired SARS-CoV-2 prior to vaccination (COVID-PreV, *n* = 74); and individuals that acquired a vaccine breakthrough infection (COVID-PostV, *n* = 35). At 2 wpv, geometric mean positivity index (GMPI) in COVID-PreV was significantly higher than that of Non-COVID and COVID-PostV groups, suggesting that prior infection boosted anti-S/RBD IgG antibody response (Fig. 3A). Then, in the Non-COVID and COVID-PreV groups, a 1.6- and 1.1-fold decrease in antibody levels between 2 and 24 wpv was observed, respectively, whereas, as expected, the COVID-PostV group showed a significant 1.2-fold increase in a-S/RBD IgG antibody levels (Fig. 3A).

### Neutralizing humoral response against ancestral SARS-CoV-2.

In order to study the neutralizing capacity against SARS-CoV-2 after Sputnik V vaccination, we selected a subgroup of 49 individuals comprising 31 Non-COVID, 9 COVID-PreV, and 9 COVID-PostV that were seropositive by COVIDAR IgG ELISA at 2 wpv (Table 1). Assessment of the neutralizing capacity against ancestral SARS-CoV-2 (Fig. 3B) indicated that 100% of individuals had detectable neutralizing antibodies at 2 wpv. At 2 wpv, neutralizing titers (NT) were similar in the two groups that had not acquired COVID-19 (Non-COVID and COVID-PostV), and significantly higher NT were obtained for previously infected individuals. At 24 wpv, 100% of the individuals exposed to infection retained neutralizing ability (COVID-PreV and COVID-PostV), whereas in the naive Non-COVID cohort, 10% of the samples fell below detectable levels. Moreover, differences between Non-COVID and COVID-PreV were still observed, with geometric mean neutralizing titers (GMNT) nearly four times lower in the Non-COVID group. Nevertheless, over time, both groups showed a decrease of nearly 2.5-fold in their NT. In turn, the COVID-PostV group showed a 5.6-fold increase in NT between 2 and 24 wpv and displayed 18 and 5 times higher NT at 24 wpv than Non-COVID and COVID-PreV groups, respectively (Fig. 3B). Overall, neutralizing antibody response paralleled the trajectory of anti-S/RBD IgG.

**TABLE 1:** Total IgG and neutralizing titers (NT) against SARS-CoV-2^*a*^

		Anti-S/RBD COVIDAR (PI)		Roche (U/mL)		NT vs ancestral		NT vs gamma		NT vs Delta	
Group	Sample ID	Wks postvaccination									
		2	24	2	24	2	24	2	24	2	24
Non-COVID	404	>10	1.3	89.9	29.0	16	12	24	1	1	1
	570	>10	1.7	50.6	52.0	8	1	12	8	1	1
	835	>10	1.9	137.0	116.0	16	8	32	8	1	1
	891	>10	2.0	ND	41.0	16	8	12	8	1	1
	574	>10	2.1	205.6	63.0	8	8	48	8	1	1
	480	>10	2.2	120	43.0	32	8	8	1	1	1
	550	>10	2.3	218	70.0	12	1	1	1	1	1
	171	>10	2.4	>250	173.0	32	12	12	1	1	1
	899	>10	3.3	137.9	87.0	12	8	8	1	1	1
	140	>10	3.5	122.4	39.0	16	1	1	1	1	1
	587	>10	4.1	165	96.0	48	16	8	1	1	1
	837	>10	4.8	>250	125.0	24	8	8	1	1	1
	367	>10	5.3	>250	159.0	64	16	12	1	1	1
	426	>10	5.8	>250	243.0	96	24	8	1	1	1
	930	>10	6.8	>250	163.0	48	24	24	8	1	1
	775	>10	7.0	200.0	107.0	12	8	1	1	1	1
	485	>10	7.5	196.0	244.0	24	32	8	16	1	8
	346	>10	8.1	>250	>250	96	32	12	12	8	8
	327	>10	9.1	>250	>250	192	96	16	24	12	16
	579	>10	9.7	>250	>250	96	24	16	8	8	1
	908	>10	>10	144.0	>250	24	192	8	128	1	24
	890	>10	>10	>250	>250	24	768	1	192	1	256
	961	>10	>10	185.0	242.0	64	64	10	8	1	1
	794	>10	>10	116.0	>250	48	64	12	8	1	8
	783	>10	>10	>250	>250	256	192	32	16	12	8
	744	>10	>10	182.0	>250	96	96	24	12	1	8
	638	>10	>10	>250	>250	192	48	32	16	1	1
	663	>10	>10	>250	>250	768	512	192	96	32	48
	675	>10	>10	>250	>250	512	256	64	48	32	16
	971	>10	>10	ND	>250	128	96	24	16	1	8
	348	>10	>10	>250	>250	512	48	24	24	12	1
COVID-PreV	395	4.2	1.8	87.0	63.0	24	8	1	1	1	1
	652	>10	3.9	71.0	103.0	16	20	8	1	1	1
	833	>10	4.5	>250	142.0	192	48	24	8	1	1
	284	>10	6.3	>250	>250	256	256	96	32	24	10
	895	>10	6.4	>250	>250	384	96	32	16	1	1
	475	>10	6.8	>250	192.0	96	32	12	8	1	1
	677	>10	>10	>250	>250	768	192	96	64	32	8
	278	>10	>10	>250	>250	768	192	32	20	32	12
	669	>10	>10	>250	>250	1024	384	128	160	128	64
COVID-PostV	478	>10	3.8	>250	>250	512	512	192	96	256	96
	40	>10	>10	177.0	>250	160	768	12	128	1	24
	561	>10	>10	>250	>250	384	192	96	24	48	16
	838	>10	>10	>250	>250	192	192	8	24	8	24
	549	>10	>10	236.0	>250	32	192	1	128	1	128
	398	>10	>10	>250	>250	16	384	8	128	1	128
	377	>10	>10	>250	ND	24	384	12	384	1	384
	82	>10	>10	>250	>250	32	1024	8	384	1	384
	927	>10	>10	156.0	>250	16	512	8	512	1	512

a Forty-nine individuals with paired samples at 2 and 24 weeks post second dose of Sputnik V were analyzed for Anti-S/RBD and neutralizing antibodies. Cohort was differentiated into individuals (i) Non-COVID (*n*= 31); (ii) COVID-PreV (*n* = 9); and (iii) COVID-PostV (*n* = 9). Anti-S/RBD IgG tested with COVIDAR (IP), Anti-S IgG tested with Roche (U/mL), and neutralizing titers (NT) against ancestral SARS-CoV-2, Gamma and Delta, are shown. Samples with NT below 8 were set at 1. ND, not determined.

### Neutralizing humoral response against SARS-CoV-2 VOCs.

**FIG 4:**
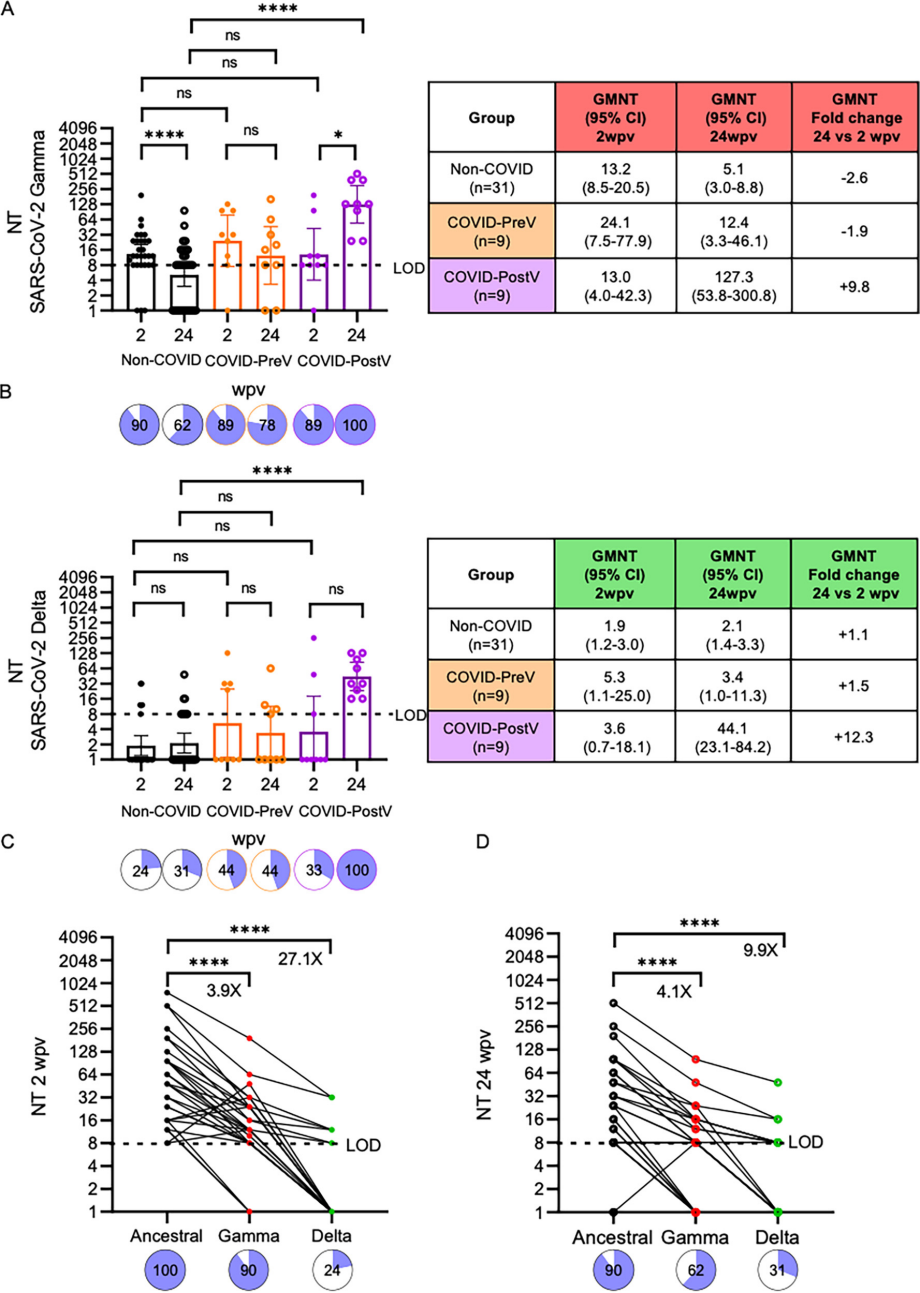
Neutralizing titers against SARS-CoV-2 Gamma and Delta VOCs after Sputnik V vaccination. Neutralizing titers (NT) were defined as the highest serum dilution without any cytopathic effect on the monolayer using a replicating SARS-CoV-2 Gamma and Delta. Sera collected at 2 and 24 weeks after completion of the vaccination schedule (wpv) with Sputnik V were used. (A and B) Cohort was differentiated into the following individuals: (i) Non-COVID, black dots (*n* = 29); (ii) COVID-PreV, orange dots (*n* = 9); and (iii) COVID-PostV, purple dots (*n* = 9). Sera were evaluated against (A) SARS-CoV-2 Gamma and (B) Delta. Individual NT values and geometric mean with 95%CI are shown. Tables next to each graph indicate the corresponding GMNT, 95%CI values, and GMNT fold change of 24 versus 2 wpv. Pie charts illustrate the percentage of samples that resulted positive for neutralizing antibodies (NT ≥ 8). (C and D) NT at (C) 2 wpv or (D) 24 wpv of sera from 29 naive individuals. NT for each sample is shown, and lines connect the same sample. Pie charts illustrate the percentage of samples that resulted positive for neutralizing antibodies (NT ≥ 8). Samples with NT below 8 (limit of detection, LOD) were set at 1 to calculate GMNTs. Wilcoxon matched-pair test was used for longitudinal analysis of the neutralizing titer. For nonpaired samples analysis, the Mann-Whitney U test was used. Statistical significance is shown with the following notations: ****, *P* < 0.0001; *, *P* < 0.05; ns, not significant.

Considering the main VOCs that circulated in Argentina from January to October 2021, the neutralizing titers against SARS-CoV-2 Gamma and Delta were also assessed (Fig. 4A and B) (Table 1). At 2 wpv, 90% of individuals in the three groups of study had detectable neutralizing antibodies against the Gamma variant. As observed for ancestral virus, neutralizing titers against Gamma were also similar between Non-COVID and COVID-PostV, and nonstatistically significant 1.8 times higher titers were obtained for the COVID-PreV group (Fig. 4A). NT against SARS-CoV-2 Gamma dropped over time in Non-COVID and COVID-PreV groups, displaying a 2.6- and 1.9-fold decrease, respectively. Breakthrough infection led to a 9.8-fold increase in NT at 24 wpv. Indeed, this group showed the highest neutralizing antibody levels, displaying 25 and 10 times higher NT than Non-COVID and COVID-PreV groups, respectively. For SARS-CoV-2 Delta (Fig. 4B), however, most of the samples presented neutralization levels below the lower dilution tested. Neutralizing antibodies were detectable in approximately 24% and 33% of individuals without previous diagnosis of COVID (Non-COVID and COVID-PostV, respectively) and in 44% of individuals with previous diagnosis (COVID-PreV). In the COVID-PostV group, exposure to the virus led to 100% positivity and to an increase in the neutralizing titers. To further study the cross-neutralizing response, we focused the analysis on the Non-COVID group (Fig. 4C and D). Neutralizing titers at 2 wpv against ancestral SARS-CoV-2 were 3.9 and 27.1 times higher than those of those against Gamma and Delta VOCs, respectively (Fig. 4C). At 24 wpv, this difference was smaller for the Delta variant, where NT were 9.9 times lower than those against ancestral virus (Fig. 4D). As observed before, the neutralizing response and the number of positive samples (NT ≥ 8) against ancestral and Gamma variant decreased between 2 and 24 wpv. For the Delta variant, NT slightly increased over time at the expense of four individuals whose neutralizing antibodies became detectable at 24 wpv. Altogether, we observed that VOCs Gamma and Delta significantly escape the neutralizing response elicited by Sputnik V vaccination and that hybrid immunity tends to confer a wider range of protection.

### Correlation of anti-Spike/RBD antibody levels and neutralizing antibody titers.

**FIG 5:**
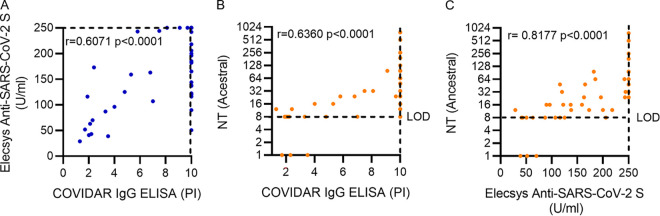
Correlation between anti-S/RBD antibodies and neutralizing antibodies against ancestral SARS-CoV-2 after Sputnik V vaccination in naive individuals. Sera of 29 naive individuals collected at 2 and 24 weeks after completion of the vaccination schedule (wpv) with Sputnik V were used. (A) Correlation between IgG anti-S/RBD (PI) tested with COVIDAR and Anti-S/RBD antibodies tested with Elecsys Anti-SARS-CoV-2 S assay (Roche) (*n* = 56). Samples with PI of ≥10 were set at PI = 10, and samples with antibody titers above 250 U/mL were set at 250. (B) Correlation of NT with IgG anti-S/RBD (PI) tested with COVIDAR IgG (*n* = 58). Samples with PI of ≥10 were set at PI = 10. (C) Correlation of NT with anti-S/RBD antibody titers tested with Elecsys assay (Roche) (*n* = 56). Samples with antibody titers above 250 U/mL were set at 250. NT below 8 were set at 1. LOD indicates the limit of detection of neutralizing antibodies. Correlation was analyzed by Spearman’s *r* test. *P* < 0.05 was considered significant.

Correlation of anti-Spike/RBD antibody levels and neutralizing antibody titers was previously reported (6 to 12). Here, we evaluated correlation in the Non-COVID group by analyzing levels of specific antibodies and neutralizing antibodies against ancestral SARS-CoV-2. Out of the 732 samples obtained at 2 wpv, 572 fell out of the dynamic range of COVIDAR assay (PI [positivity index] > 10), including the samples selected for paired analysis of neutralization in naive individuals (*n* = 29). This suggests that this assay offers a limited dynamic range to discriminate samples with high antibody titers. Therefore, for the purpose of the correlation analysis, we also determined total anti-S antibody levels of the paired samples of the 29 individuals in the Non-COVID group (*n* = 56) using electrochemiluminescence Elecsys Anti-SARS-CoV-2 S assay (Roche) (Fig. 5). For the paired samples selected for analysis, only 31% fell in the dynamic range (PI < 10) of COVIDAR, while using Elecsys, 52% fell in the dynamic range. As a result, antibody levels determined with COVIDAR and Elecsys assays showed a modest correlation (*r* = 0.6071) (Fig. 5A). Next, we evaluated the correlation between NT and PI obtained by the COVIDAR IgG ELISA. We observed that using this test, anti-S/RBD IgG levels correlated with NT (*r* = 0.6360) (Fig. 5B). In turn, the analysis of samples tested with Elecsys Anti-SARS-CoV-2 S assay showed a robust correlation (*r* = 0.8177) between total anti-S/RBD and NT (Fig. 5C). Altogether, the results indicate that total IgG levels correlate with NT. Differences in the strength of correlation obtained when NT were compared with antibody levels determined using COVIDAR or Elecsys assays point to the broader dynamic range offered by Elecsys to discriminate antibody levels between samples selected for analysis.

## DISCUSSION

This work presents a comprehensive 6-month follow-up study of the humoral response of a cohort of health care workers vaccinated with Sputnik V from December 2020 to March 2021. Our results add evidence to the response to vaccination with Sputnik V in the medium term, and report for the first time the follow-up of the neutralizing antibody response against ancestral SARS-CoV-2 and VOCs according to a differentiated analysis of naive and COVID-diagnosed individuals.

In accordance with previous reports, our results clearly reflect that the Sputnik V vaccine induces a robust humoral response against SARS-CoV-2 that is enhanced with a second dose (1, 2, 6, 10, 11, 18, 19). Moreover, our data confirm improved response to vaccination of previously infected individuals. Accordingly, the IgG antibody levels after a single dose of vaccine in individuals with previous infection were similar to those obtained after two doses of vaccine in naive individuals (data not shown), and after completing the vaccination schedule, anti-S/RBD IgG levels were also higher in those individuals who were previously infected. These results indicate that naturally acquired immunity is enhanced by one or two doses of vaccine (7, 11, 16, 18, 20 to 24) and reinforce what was previously reported not only for Sputnik V but also for other vaccine platforms (6, 7, 10, 16, 18 to 20, 25). Similarly, our longitudinal analysis indicates that individuals vaccinated with Sputnik V maintain robust antibody levels after 6 months, contributing additional evidence to the persistence of the humoral response induced by vaccination over time (6, 7, 9, 10, 19). Notwithstanding, we observed a decrease in antibody levels both in naive and in previously infected individuals (6, 7).

Altogether, our study design revealed that hybrid immunity in the context of Sputnik V vaccination led to an enhancement of the humoral response: individuals with a previous exposure to the virus had the highest antibody response after vaccination (2 wpv) and tended to retain higher antibody levels than naive individuals even up to 6 months (24 wpv). On the other hand, individuals with a breakthrough infection after vaccination had the highest antibody levels at 24 wpv.

Analysis of the neutralizing antibody response in a subgroup of 49 individuals receiving the complete Sputnik V vaccination scheme indicated that neutralizing response against ancestral SARS-CoV-2 paralleled total IgG seroconversion and persistence, and 100% of seropositive individuals had detectable neutralizing antibodies.

Neutralizing titers were in line with anti-S/RBD levels: neutralization potency was higher in those individuals who were previously infected and decreased after 6 months in naive individuals (9, 10, 16, 18 to 20, 25). Interestingly, we also demonstrated that, although previously infected individuals maintained higher NT than naive after 6 months of vaccination, their neutralization potency also decreased. This suggests that waning of the humoral response is a common feature following infection or vaccination, and that humoral response is enhanced by a new exposure (infection or vaccine boost), as reflected here by the 5-fold increment in NT in individuals with a breakthrough infection.

Our seroprevalence study coincided with cocirculation of the Alpha, Lambda, and Gamma VOCs along with other variants. Gamma became the most prevalent from mid-April. Since June 2021, the Delta variant began to circulate, becoming the most prevalent in September of that year, being together with Gamma the main variants causing the second wave of COVID-19 in Argentina (26). Like the other first generation of COVID-19 vaccines, Sputnik V is based on the Wuhan SARS-CoV-2 spike sequence, and it was especially relevant to evaluate the cross-neutralization capacity of antibodies against SARS-CoV-2 Gamma and Delta in vaccinated individuals. As expected, the potency of neutralization against VOCs was smaller than against the ancestral virus (9, 10, 16, 19, 25). Despite this difference, 90% of individuals presented cross-neutralizing antibodies against Gamma after 2 doses of Sputnik V, regardless of whether they were previously infected or not. Noteworthy, detection of neutralizing antibodies against Delta markedly differed between naive and previously infected individuals. While 44% of individuals acquiring infection prior to vaccination presented neutralizing antibodies, less than 20% of the vaccinated individuals who were naive at 2 wpv were positive. Although the number of individuals in our study is limited, the analysis of cross-reactivity reinforces the concept that hybrid immunity generates a broader immune response (14, 15). In line with this notion, after 6 months of vaccination, 100% of those individuals who had COVID-19 between 2 and 24 wpv had detectable neutralizing antibodies against both VOCs. This may be explained not only by hybrid immunity but also by the fact that these individuals may have been infected by Gamma or Delta VOCs, and infection with VOCs after vaccination may have also potentiated the immune response against ancestral virus and heterologous VOCs (25). In conclusion, vaccination reinforces and is reinforced by infection where hybrid immunity may play a key role in cross protection.

To gain insight into whether antibody levels against S or RBD, usually determined in the clinical settings, are good predictors of neutralizing response, we analyzed the correlation between specific antibody levels and neutralizing titers obtained at 2 and 24 wpv in the Non-COVID cohort. Specific antibody levels were assessed with a semiquantitative ELISA (COVIDAR IgG) and a quantitative electrochemiluminescence assay with a broader dynamic range (Elecsys Anti-SARS-CoV-2 S). The analysis indicated that specific antibody levels against S/RBD correlated with NT against ancestral virus for both semiquantitative and quantitative techniques. This correlation was previously noted in convalescent and vaccinated individuals (24, 27 to 32). For COVIDAR IgG, correlation was reported for convalescent-phase samples (33), our work being the first report showing this correlation in samples of vaccinated individuals.

Altogether, our data and previous reports indicate that the humoral response elicited by vaccination with Sputnik V shares common features with vaccines based on different platforms. Similar to mRNA vaccines, response after vaccination persisted over time despite waning of total IgG and neutralizing antibody titers. The magnitude of the drop is comparable for both adenovirus and mRNA-based vaccines and was is in the range of 2- to 10-fold decrease between peaking of antibody response (2 to 3 weeks after completion of the vaccination schemes) and 6 months (24, 34, 35). In addition, antibody titers are induced to a similar higher extent in individuals that acquired COVID-19 prior to vaccination compared to naive individuals (24, 34), and breakthrough infection boosts the antibody response for both platforms (12, 14, 15, 36). Finally, partial escape to vaccine-induced antibodies of VOCs Gamma and Delta as assessed by the fold reduction in neutralization titers is also a common feature (25) and suggests that the continued emergence of VOCs impacts the breadth of protection conferred by vaccination.

Overall, our work provides comprehensive information regarding the response induced by Sputnik V vaccination by means of an analysis that comprised more than 1,100 individuals and the follow-up of more than 500 fully vaccinated individuals. We showed that humoral/neutralizing response persists after Sputnik V vaccination and that neutralizing capacity decreases over time if no further immunization (natural or by a vaccine boost) may occur (12), highlighting the importance of administering a complete scheme and booster doses in the medium term to keep neutralizing titers both in naive and vaccinated-infected individuals. Likewise, Sputnik V has been approved for use in 71 countries, including several countries in Latin America, and although the global political context has paralyzed the verification by the WHO of the efficacy of this vaccine, our work adds new clear and necessary evidence regarding the response mounted by Sputnik V.

## MATERIALS AND METHODS

### Ethics statement.

This study was approved by the Ethics Committee at the Facultad de Ciencias Bioquímicas y Farmacéuticas-UNR, Rosario, Santa Fe, Argentina (res. no. 620/2021). All participants provided written informed consent prior to collection of data and specimen.

### Samples and SARS-CoV-2 antibody ELISA.

Blood serum samples were obtained by venipuncture and stored at −20°C. Antibodies to SARS-CoV-2 spike protein (S) and receptor binding domain (RBD) were detected using the COVIDAR IgG ELISA (Laboratorio LEMOS, S.R.L, Buenos Aires, Argentina) (33). Antibody levels were given by a positivity index (PI), which varied between 1 and 10. PI results from the ratio between the absorbance of the sample and the cutoff value of the methodology. This cutoff value was calculated following the manufacturer’s instruction as (absorbance of negative control + 0.2) × 1.1.

To study the correlation between neutralizing capacity against SARS-CoV-2 and antibodies against S or RBD in the cohort of naive individuals, Elecsys Anti-SARS-CoV-2 S assay (Roche Diagnostics, Mannheim, Germany) was also used. This electrochemiluminescence immunoassay targets the RBD with an analytical measuring interval between 0.4 and 250.0 U/mL.

### Cells and virus.

Vero E6 cells (ATCC CRL-1586) were cultured at 37°C and 5% CO_2_ in complete Dulbecco’s modified Eagle’s medium (DMEM, Gibco) containing 10% fetal bovine serum (FBS) (Internegocios, Mercedes, Buenos Aires, Argentina), 100 IU/mL penicillin, and 100 μg/mL streptomycin (Gibco). Ancestral SARS-CoV-2 (B.1 with the D614G substitution, GISAID accession ID EPI_ISL_15806335) and VOC Gamma (P.1, GISAID accession ID EPI_ISL_15807444) were isolated from nasopharyngeal specimens at the IIBIO (Universidad Nacional de San Martín) and adapted in Vero E6 cultures. VOC Delta (B.1.617.2, GISAID accession ID: EPI_ISL_11014871) was isolated at INBIRS (Facultad de Medicina, UBA-CONICET, Argentina). Virus isolates sequences are available on GISAID and accesible at https://doi.org/10.55876/gis8.230406sp.

### Neutralization assay.

Vero E6 cells were seeded in 96-well plates at a density of 1.5 × 10^4^ cells per well in complete DMEM and incubated 24 h at 37°C and 5% CO_2_. SARS-CoV-2 (300 TCID50) was preincubated with serially diluted sera for 1 h at 37°C starting at sera dilution 1:8. Each sera dilution was tested in duplicate. Then, virus–sera mixture was added onto Vero E6 cells in a final volume of 100 μL in DMEM 2% FBS. After 72 h at 37°C and 5% CO_2_, cultures were fixed with formaldehyde 10% at 4°C for 24 h and stained with crystal violet solution in methanol. The cytopathic effect (CPE) of the virus on the cell monolayer was assessed visually, and neutralization titer (NT) was defined as the inverse of the highest serum dilution without any CPE.

### Statistical analysis.

GraphPad version 8.4.2 was used for statistical analyses and generation of plots. The geometric means (GM) and 95% CI were calculated in each group of samples. Samples with NT below 8 were set at 1 to calculate GMNT and to represent them in the corresponding plots. After normality and lognormality tests, nonparametric tests were used. Paired data were analyzed by Wilcoxon matched-pair test, nonpaired data by the Mann-Whitney U test and Kruskal-Wallis test. Correlation between total antibody levels and neutralizing titers was analyzed by Spearman’s *r* test. Statistical significance is shown with the following notations: ****, *P* < 0.0001; ***, *P* < 0.001; **, *P* < 0.01; *, *P* < 0.05; ns, not significant.

### Data sharing.

Data used in this study are available upon request from the corresponding author.

### Ethics approval.

This study was approved by the Ethics Committee at the Facultad de Ciencias Bioquímicas y Farmacéuticas-UNR, Rosario, Santa Fe, Argentina (res. no. 620/2021).
